# Seaweed Polysaccharides and Derived Oligosaccharides Stimulate Defense Responses and Protection Against Pathogens in Plants

**DOI:** 10.3390/md9122514

**Published:** 2011-11-29

**Authors:** Jeannette Vera, Jorge Castro, Alberto Gonzalez, Alejandra Moenne

**Affiliations:** Laboratory of Marine Biotechnology, Faculty of Chemistry and Biology, University of Santiago, Santiago 9160000, Chile; Email: jvera22@gmail.com (J.V.); castroponce78@gmail.com (J.C.); alberto.gonzalezfi@usach.cl (A.G.)

**Keywords:** seaweeds, oligosaccharides, ulvans, alginates, fucans, laminarin, carrageenans, defense responses, terrestrial plants

## Abstract

Plants interact with the environment by sensing “non-self” molecules called elicitors derived from pathogens or other sources. These molecules bind to specific receptors located in the plasma membrane and trigger defense responses leading to protection against pathogens. In particular, it has been shown that cell wall and storage polysaccharides from green, brown and red seaweeds (marine macroalgae) corresponding to ulvans, alginates, fucans, laminarin and carrageenans can trigger defense responses in plants enhancing protection against pathogens. In addition, oligosaccharides obtained by depolymerization of seaweed polysaccharides also induce protection against viral, fungal and bacterial infections in plants. In particular, most seaweed polysaccharides and derived oligosaccharides trigger an initial oxidative burst at local level and the activation of salicylic (SA), jasmonic acid (JA) and/or ethylene signaling pathways at systemic level. The activation of these signaling pathways leads to an increased expression of genes encoding: (i) Pathogenesis-Related (PR) proteins with antifungal and antibacterial activities; (ii) defense enzymes such as pheylalanine ammonia lyase (PAL) and lipoxygenase (LOX) which determine accumulation of phenylpropanoid compounds (PPCs) and oxylipins with antiviral, antifugal and antibacterial activities and iii) enzymes involved in synthesis of terpenes, terpenoids and/or alkaloids having antimicrobial activities. Thus, seaweed polysaccharides and their derived oligosaccharides induced the accumulation of proteins and compounds with antimicrobial activities that determine, at least in part, the enhanced protection against pathogens in plants.

## 1. Introduction

The principal cell wall polysaccharides in green seaweeds are ulvans, those in red seaweeds are agarans and carrageenans and those in brown seaweeds are alginates and fucans as well as the storage polysaccharide laminarin [1,2]. The elicitor activities of these polysaccharides and derived oligosaccharides were initially described by Patier *et al*. using extracts of the brown seaweed *Ascophyllum nodosum* and *Rubus fruticosus* cell suspensions [3] and by Patier *et al*. using depolymerized kappa carrageenan and *Rubus* cells and protoplasts [4]. The *Ascophyllum* extract and kappa oligo-carrageenan induced an increase in activity of the defense enzyme β-1,3-glucanase having antifungal properties. It has since been shown that most seaweed polysaccharides and derived oligosaccharides activate defense responses and protection against a broad range of pathogens in terrestrial plants. 

## 2. Structure of Seaweed Polysaccharides and Derived Oligosaccharides

### 2.1. Ulvans and Oligo-Ulvans

**Figure 1:**
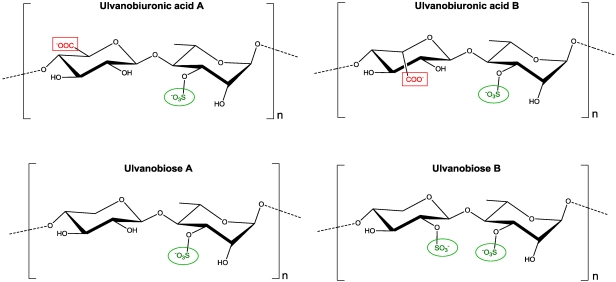
Disaccharide units of ulvabiouronic acid and ulvabiose in ulvans of green seaweeds.

Ulvans are the major the major constituents of green seaweeds cell walls representing 8 to 29% of the algal dry weight [5]. They are ramified acidic and sulphated polysaccharides constituted by a central backbone of disaccharide units formed by an L-rhamnose 3-sulphate linked to: (i) a D-guluronic acid residue (ulvabiouronic acid unit A); (ii) a L-iduronic acid residue (ulvabiuronic acid unit B); (iii) a D-xylose 4-sulphate residue (ulvabiose unit A); or (iv) a D-xylose residue (ulvabiose unit B) (Figure 1). In addition, ulvans show ramifications in position O-2 of the rhamnose 3-sulphate residue. On the other hand, oligo-ulvans have been obtained by depolymerization of cell wall polysaccharides from *Ulva armoricana*, *U. rigida*, *U. lactuca*, *U. compressa* and *U. intestinalis* using 2 M HCl at 100 °C for 45 min which produces mainly monosaccharide and disaccharide units [6,7]. In addition, oligo-ulvans with a molecular weight of 50 to 60 kDa have been obtained by ultrasound fragmentation and further purification by size exclusion chromatography [8].

### 2.2. Alginates and Oligo-Alginates

**Figure 2:**
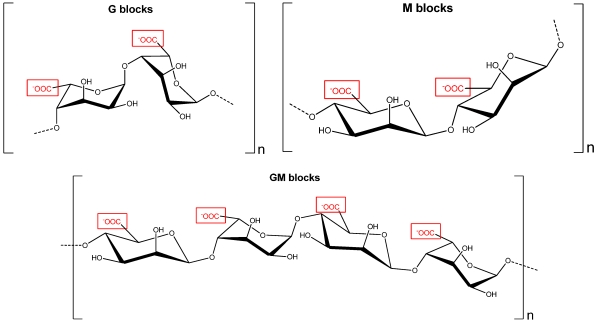
Units of poly D-glucuronic acid (G blocks), poly D-mannuronic acid (M blocks) and alternate D-glucuronic and D-mannuronic acid (GM blocks) in alginates of brown seaweeds.

Alginates are the major constituent of brown seaweeds cell walls representing 17 to 45% of the algal dry weight [9]. They are linear acidic polysaccharides constituted by a central backbone of poly-D-glucuronic acid (G blocks), poly-D-mannuronic acid (M blocks) and alternate residues of D-guluronic acid and D-mannuronic acid (GM blocks) (Figure 2). On the other hand, oligo-alginates with an average molecular weight of 3.5 kDa formed by D-glucuronic acid (Poly-Gu) or D-mannuronic acid (Poly-Ma) have been obtained by acid hydrolysis of G and M fractions of alginates extracted from *Lessonia trabeculata* and *L. vadosa* [10,11]. 

### 2.3. Fucans and Oligo-Fucans

**Figure 3:**
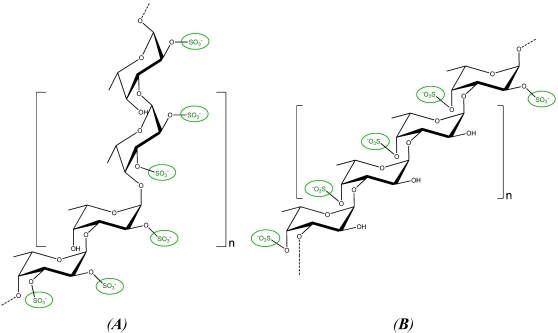
Units of sulphated fucose in fucans of *A. nodosum* (**A**) and *L. vadosa* (**B**).

Fucans are one of the major constituent of brown seaweeds cell walls representing 5 to 20% of the algal dry weight [9,12]. They are ramified sulphated polysaccharides constituted by a central backbone of fucose sulphated in positions C2 and/or C4 and ramifications at each two or three fucose residues. In particular, fucans from *Ascophyllum nodosum* and *Fucus vesiculosus* are constituted by a disaccharide unit of fucose sulphated in C2 linked to a fucose sulphated in C2 and C4 [13] (Figure 3A). In contrast, fucans from *Lessonia vadosa* are formed by fucose sulphated in C4 linked to fucose residues sulphated in C2 and C4 [14] (Figure 3B). On the other hand, oligo-fucans with an average molecular weight of 10 kDa have been obtained by enzyme digestion of fucans from *Pelvetia caniculata* [15] and oligo-fucans with a molecular weight of 32 kDa were prepared by free radical depolymeryzation of fucans from *Lessonia vadosa* [14].

### 2.4. Laminarin

**Figure 4:**
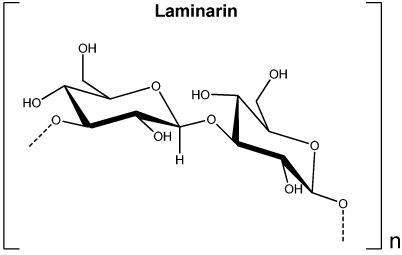
Units of glucose in laminarin of brown seaweeds.

Laminarin is the principal storage polysaccharide of brown seaweeds representing up to 35% of the algal dry weight [16]. It is mainly a linear polysaccharide constituted by 25 to 50 glucose units linked by β-1,3-glycosidic bonds and in some cases having β-1,6-glycosidic bonds and ramifications in O-6 position [2,17] (Figure 4). Laminarins have an average molecular weight of 5 kDa but they can differ in the terminal reducing end corresponding to a glucose residue in G-type laminarin and to a mannitol residue in M-type laminarin [2,17]. 

### 2.5. Carrageenans and Oligo-Carrageenans

**Figure 5:**
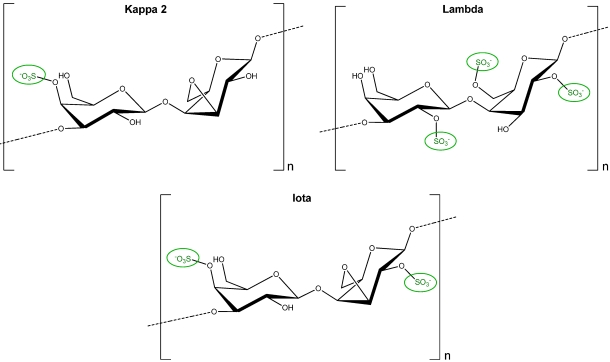
Units of sulphated D-galactose and anhydrogalactose in kappa, lambda and iota carrageenans of red seaweeds.

Carrageenans are one of the major constituents of red seaweed cell walls representing 30 to 75% of the algal dry weight [18]. They are linear sulphated polysaccharides constituted by a central backbone of D-galactose sulphated in different positions linked to anhydrogalactose units in some cases. In particular, kappa carrageenan is constituted by a D-galactose sulphated in C4 linked to anhydrogalactose, lambda carrageenan is formed by a D-galactose sulphated in C2 linked to a D-galactose sulphated in C2 and C6, and iota carrageenan is constituted by a galactose sulphated in C4 linked to an anhydrogalactose sulphated in C2 (Figure 5). Thus, the amount of sulphate groups is higher in lambda carrageenan followed by iota carrageenan and then kappa carrageenan and only kappa and iota carrageenans contain anhydrogalactose residues. On the other hand, oligo-carrageenans with an average molecular weight of 10 kDa have been obtained by acid hydrolysis of kappa, lambda and iota carrageenans using 0.1 N HCl at 60 °C for 45 min [19]. 

## 3. Polysaccharides and Derived Oligosaccharides Stimulate Defense Responses and Protection Against Pathogens

### 3.1. Ulvans and Oligo-Ulvans

Ulvans extracted from *U. fasciata* did not induce an oxidative burst in rice and wheat cells at a concentration of 0.2 mg·mL^−1^ but they showed a priming effect reflected on the amplification of the oxidative burst induced by a chitin oligosaccharide or a chitosan polysaccharide [20]. In addition, ulvans at a concentration of 1 mg·mL^−1^ induced an increase in expression of genes encoding enzymes of the phenylpropanoid pathway such as phenylalanine ammonia lyase (PAL), chalcone synthase (CHS), chalcone isomerase (CHI), chalcone reductase (CHR), caffeic acid *O*-methyltransferase (CMT) and isoflavone reductase (IFR) in *Medicago truncatula* plants [21]. Moreover, ulvans induced an increase in expression of genes involved in the octadecanoid defense pathway such as lipoxygenase (LOX), oxophytodienoate reductase and a fatty acid desaturase [6,21] as well as in expression of genes encoding PR-1 and PR-10 proteins in *M. truncatula* [6,21]. Furthermore, ulvans at a concentration of 1 mg·mL^−1^ induced a transient increase in jasmonic acid (JA) level with a maximal level at 2 h of treatment, a decrease in abscicic acid (ABA) level and no change in salicylic acid (SA) level in *M. truncatula* [8]. Furthermore, ulvans activate expression of JA-responsive genes in *Arabidopsis thaliana* suggesting that their effect involves the activation of the JA signaling pathway [7]. On the other hand, oligo-ulvans having a molecular weight of 50 to 60 kDa at a concentration of 1 mg·mL^−1^ induced a similar effect as ulvans in *M. truncatula* plants [8]. Thus, ulvans and oligo-ulvans stimulate plant defense responses probably by activation of the JA acid signaling pathway.

Consistently with the activation of defense responses, ulvans sprayed on leaves at a concentration of 1 mg·mL^−1^, one or two times in total, induced protection against the fungi *Colletotrichum trifolii* in *M. truncatula* plants [21], *Plasmopara viticola* and *Erisyphe necator* in grapevine plants [8,22], *Blumeria graminis* in wheat and barley plants [19] and *Colletotrichum lindemuthianum* in bean seedlings [23]. In addition, ulvans sprayed on leaves at a concentration ranging from 0.1 to 10 mg·mL^−1^, one or two times in total, induced protection against the bacterium *Pseudomonas syringae* in tomato plants [8], the acarid *Tetranichus urticae* in strawberry plants [8] and the nematode *Meloidogyne incognita* in tomato plants [8]. Thus, ulvans induced protection against a broad range of pathogens in plants.

### 3.2. The Oligo-Alginate Poly-Ma

Oligo-alginates constituted by D-guluronic acid (Poly-Gu, 3.5 kDa) or D-mannuronic (Poly-Ma, 3.5 kDa) prepared by acid hydrolysis and injected in wheat leaves at a concentration of 0.5 mg·mL^−1^ showed that only Poly-Ma induced an increase in PAL activity at 24 h of treatment [24]. Moreover, Poly-Gu and Poly-Ma sprayed on tobacco leaves at a concentration of 0.5 mg·mL^−1^, once a week, four times in total showed that only Poly-Ma induced a sustained increase in PAL activity [11]. In contrast, Poly-Ma and Poly-Gu did not induce an increase LOX activity in tobacco plants. These results indicate that only Poly-Ma activate defense responses in plants probably through the SA signaling pathway. Consistently with the activation of defense responses, Poly-Ma induced an effective protection against tobacco mosaic virus (TMV) in tobacco plants [11]. 

### 3.3. Fucans and Oligo-Fucans

Oligo-fucans (10 kDa) obtained by enzyme digestion of fucans at a concentration of 0.2 mg·mL^−1^ induced the release of hydrogen peroxide in tobacco cells (cv. Bright Yellow) with a maximal level at 6 min of treatment [15]. In addition, they induced a transient increase in PAL activity with a maximal level at 4 h of treatment and a sustained increase in LOX activity that remained unchanged until 20 h of treatment [15]. Moreover, oligo-fucans sprayed on tobacco leaves at a concentration of 0.2 mg·mL^−1^ induced the accumulation of PR1, PR2 (glucanase), PR3 (chitinase) and PR5 proteins [15] having antifungal and antibacterial activities [25] with a maximal level at 48 h of treatment as well as the accumulation of scopoletin, a PPC with antiviral activity [26]. Furthermore, they induced an increase in SA concentration at systemic level and an enhanced protection against TMV infection [15]. In addition, oligo-fucans (32 kDa) obtained by free radical depolymerization at a concentration of 0.5 mg·mL^−1^, sprayed on tobacco leaves, once a week, four times in total, induce an increase in PAL and LOX activities after 7 days of treatment [14]. Interestingly, the native fucan of *L. vadosa*, at a concentration of 0.5 mg·mL^−1^, induced a similar increase in PAL and LOX activities compare to oligo-fucans [14]. Thus, fucans and oligo-fucans stimulate defense responses probably by activation of SA and JA signaling pathways and induce an increased protection against TMV in tobacco plants.

### 3.4. Laminarin

Laminarin extracted from the brown seaweed *Laminaria digitata* at a concentration of 0.2 mg·mL^−1^ induced the release of hydrogen peroxide in tobacco cells (cv. Bright Yellow) with a maximal level at 5 min of treatment [27]. In addition, it induced a transient increase in PAL activity with a maximal level at 4 h, a sustained increase in LOX activity up to 20 h and the accumulation of PR-1, PR-2 (glucanase), PR-3 (chitinase) and PR-5 at 48 h of treatment [27]. Furthermore, laminarin injected in tobacco plants induced protection against the bacteria *Erwinia carotovora* infection by reducing the diameter of the necrotic lesion [27]. Thus, laminarin induced the activation of JA signaling pathway and protection against pathogens in tobacco plants. On the other hand, laminarin at 1 mg·mL^−1^ induced the release of hydrogen peroxide in grapevine cells (cv. Gamay) with a maximal level at 20 min of treatment [28]. In addition, it induced a transient increase in PAL and LOX transcripts in grapevine cells with maximal levels at 5–10 h and 2 h, respectively, a transient accumulation of PPCs, mainly reverastrol and its dimer ε-viniferin, with a maximal level at 24 h and a sustained increase in glucanase and chitinase activities up to 48 h of treatment [28]. Moreover, detached grapevine leaves incubated with laminarin at a concentration of 1 mg·mL^−1^ for 24 h and infected with the fungus *Botrytis cinerea* showed a decrease in the diameter of necrotic lesions. In addition, grapevine plants sprayed on leaves with laminarin showed protection against the fungus *Plasmopara viticola* [28]. Thus, laminarin activates SA defense pathways in grapevine plants as well as the accumulation of PR proteins and PPCs with antimicrobial activities leading to an enhanced protection against bacterial and fungal infections. 

On the other hand, laminarin sulphated with a mixture of sulphite/pyridine at 60 °C showed a complete sulphatation in C6 position and a partial sulphatation (70%) in C4 and C3 positions [29]. In particular, sulphated laminarin (PS3) and laminarin at a concentration of 0.2 mg·mL^−1^ induced the release of hydrogen peroxide in tobacco cells (var. Xanthi), PS3 induced a maximal increase at 30 to 60 min of treatment whereas laminarin showed a peak at 15 min of treatment suggesting that these polysaccharides are differentially sensed by tobacco cells [29]. In addition, the increase in extracellular hydrogen peroxide induced by PS3 was dependent on extracellular calcium entry since the oxidative burst was inhibited by EGTA and lanthanum ions. In addition, PS3 induced an increase in hydrogen peroxide that involves the activation of calcium-dependent protein kinases (CDPKs) because hydrogen peroxide production was partially inhibited by staurosporine, an inhibitor of CDPKs. In contrast, the oxidative burst induced by laminarin was completely inhibited by staurosporine suggesting that laminarin and PS3 trigger different signaling pathways [29]. Furthermore, only PS3 induced an increase in salicylic acid level and the accumulation of the PPC scopoletin in tobacco leaves. Interestingly, the increase in the degree of sulphatation in PS3 determines the increase in expression of PR proteins having antipathogenic effects [29]. In addition, transgenic tobacco and *A. thaliana* plants impaired in the accumulation of SA and ethylene treated with PS3 showed tha activation of SA- and ethylene-dependent signaling pathways whereas laminarin induced only the ethylene-dependent pathway. Furthermore, PS3 protected tobacco plants against TMV infection since no necrotic lesions developed in infiltrated leaves whereas laminarin induced only a decrease in the number of necrotic lesions [29]. Thus, PS3 triggers different signaling pathways compare to laminarin and a higher protection against TMV infection in tobacco plants. Furthermore, grapevine plants treated with PS3 showed the activation of the JA-dependent signaling pathway and an increased protection against the fungus *P. viticola* [30]. Thus, PS3 induce SA, JA and ethylene signaling pathways in different terrestrial plants and a wide protection against pathogens.

### 3.5. Carrageenans and Oligo-Carrageenans

The native lambda carrageenan infiltrated in tobacco leaves at a concentration from 0.1 to 1 mg·mL^−1^ induced an increased expression of genes coding for: (i) a sequiterpene cyclase involved in the synthesis of the antimicrobial terpenoid capsidiol; (ii) PR-3, a basic chitinase with antifungal activity; and (iii) a type II proteinase inhibitor with antipathogenic activity [31]. In addition, lambda carrageenan induced the accumulation of SA in distant leaves after 7 days of treatment indicating that the stimulation of defense responses may involve SA signaling pathway. Furthermore, lambda carrageenan induced only a transient increase in expression of genes encoding LOX and 1-aminocyclopropane-1-carboxylic acid oxidase transcripts suggesting that JA and ethylene are not involved in the activation of defense responses [31]. On the other hand, the native iota carrageenan at a concentration of 1 mg·mL^−1^ did not protect against TMV infection whereas iota oligo-carrageenan at the same concentration reduced in 79% the number of necrotic lesions in tobacco plants [32] indicating that the depolymerization of carrageenans is strictly required to induce an effective protection against pathogens in tobacco plants. The latter contrasts with results obtained with ulvans/oligo-ulvans and fucans/oligo-fucans where the native polysaccharides and the derived oligosaccharides induced similar defense responses.

In addition, a depolymerized fraction of the native sulphated galactan from the red alga *Schizimenia binderi* (Poly-Ga), an oligosaccharide structurally related to lambda oligo-carrageenan, sprayed on leaves of tobacco plants (var. Xanthi) at a concentration of 0.5 mg mL^−1^, once a week, four times in total, induced a sustained activation of PAL defense enzyme whereas no increase in LOX activity [33]. Interestingly, increasing concentrations of Poly-Ga, increasing number of treatments and increasing time after treatment enhanced protection against TMV infection in tobacco plants indicating that Poly-Ga induced a long-term protection that mimicks vaccination [33]. In addition tobacco plants treated with Poly-Ga without infection showed a sustained activation of PAL enzyme, even 45 days after treatment, and the accumulation of several phenylpropanoid compounds (PPCs) with antimicrobial activities. The sustained activation of PAL and the accumulation of PPCs may explain, at least in part, the enhanced protection against TMV infection induced by Poly-Ga in tobacco plants [33].

On the other hand, oligo-carrageenans kappa, lambda and iota obtained by acid hydrolysis of commercial carrageenans [19] also induced a long-term protection against TMV infection that mimics vaccination in tobacco plants (var. Xanthi) [34]. In addition, tobacco plants treated with kappa, lambda and iota oligo-carrageenans at a concentration of 1 mg·mL^−1^, once a week, three times in total and cultivated for 45 days showed protection against TMV, *B. cinerera* and *E. carotovora* infections indicating that oligo-carrageenans induced a broad range protection against pathogens. In particular, the highest protection against TMV was obtained with lambda oligo-carrageenan, that against *B. cinerea* was observed with lambda and iota oligo-carrageenans and protection against *E. carotovora* was similar with the three oligo-carrageenans [34]. Thus, oligo-carrageenans induced a long-term and broad-range protection against pathogens in tobacco plants. Interestingly, oligo-carrageenans kappa, lambda and iota also induced the reversion of viral, fungal and bacterial infections at systemic level with a high efficiency for *B. cinerea* and *E. carotovora* infections and a moderate efficiency (around 50% inhibition) for TMV infection in tobacco plants [34]. 

In addition, tobacco plants treated with oligo-carrageenans kappa, lambda and iota at a concentration of 1 mg·mL^−1^ and cultivated for 45 days without infection showed a sustained increase in PAL activity whereas no increase in LOX activity. The increase in PAL activity in plants treated with oligo-carrageenans kappa, lambda and iota leads to a similar accumulation of SA but to a differential accumulation of PPCs. In particular, tobacco plants treated with oligo-carrageenans kappa accumulated mainly dehydrobenzoic acid, gallic acid and vainillic acid whereas plants treated with oligo-carrageenan iota accumulated mainly caffeic acid, chlorogenic acid and esculetin [34]. Thus, oligo-carrageenans kappa, lambda and iota induced a sustained activation of PAL, a similar accumulation of SA and a different accumulation of PPCs with antimicrobial activities which may determine, at least in part, the enhanced protection against a broad range of pathogens. 

Finally, it is important to mention that oligo-carrageenans kappa, lambda and iota stimulate growth in tobacco plants by enhancing photosynthesis, basal metabolism and cell division, mainly oligo-carrageenan iota [35,36]. In addition, oligo-carrageenans stimulate growth of eucalyptus trees by enhancing photosynthesis and basal metabolism, mainly oligo-carrageenan kappa [37]. Thus, oligo-carrageenans induced a dual beneficial effect in terrestrial plants corresponding to an enhanced protection against pathogens and to the stimulation of plant growth.

## 4. Conclusions

In summary, seaweeds polysaccharides such as ulvans, alginates, fucans, laminarin and carrageenans and their derived oligosaccharides can induce an initial oxidative burst and the activation of SA, JA and/or ethylene signaling pathways in terrestrial plants. The activation of these signaling pathways leads to an increase in expression of PR proteins having antifungal and antibacterial activities and to an increased expression of defense enzymes that participate in the synthesis of PPCs, terpenes, terpenoids and alkaloids with antimicrobial activities. Thus, the accumulation of proteins and compounds with antimicrobial activities may determine, at least in part, the enhanced protection against pathogens induced by seaweed polysaccharides and their derived oligosaccharides in terrestrial plants.
